# Reimagining Israel’s food system: balancing mediterranean diet recommendations with national food security, sovereignty and resilience

**DOI:** 10.1186/s13584-026-00748-1

**Published:** 2026-03-06

**Authors:** Ella Berkovich, Stav Shapira, Moran Koren, Nimrod Talmon, Dorit Nitzan

**Affiliations:** 1 School of Public Health, Ben-Gurion University of the Negev, Beer Sheva, Israel; 2 Department of Industrial Engineering and Management, Ben Gurion University of the Negev, Beer Sheva, Israel

**Keywords:** Mediterranean Diet (MD), Food Security, Import Dependency (IDR), Food Sovereignty, Food Systems Resilience, Sustainable Nutrition, Food Balance Sheets (FBS), One Health Approach

## Abstract

**Background:**

This study examines Israel’s food system’s security, sovereignty, resilience, and sustainability using the Mediterranean Diet (MD) lens. The current global context, marked by supply chain disruptions, climate change impacts, and geopolitical tensions, highlights the critical importance of resilient food systems. We analyzed Israel’s food system using FAO Food Balance Sheets (FBS) (2010–2020 trends) and Israel Central Bureau of Statistics (CBS) production and trade data (2021) to assess food availability, import dependency ratios (IDR), and alignment with Mediterranean Diet guidelines.

**Methods:**

A literature search was conducted using PubMed, Semantic Scholar, EBSCO Discovery, and Google Scholar, aiming to agree on the food-based guidelines of MD and their impact on health outcomes. Studies from the past 10 years with the keywords “Mediterranean diet” and “meta-analysis” were included. The Israeli agricultural production data from 2010 to 2020 were drawn from the Central Bureau of Statistics (CBS). Import dependence was calculated by converting product quantities from tonnes to grams per person per day (annual quantity × 10 million people ÷ 365 days).

**Results:**

Israel demonstrates significant import dependency across critical food groups, with 90% of cereals, 87% of fish, 82% of nuts, and 65% of added fats sourced internationally. While nearly self-sufficient in poultry and dairy, chickens and cows grown in Israel depend on imported feed. Moreover, the country’s food supply significantly deviates from the MD recommendations. Notably, there is an undersupply of nature-based proteins, including legumes.

**Conclusion:**

The results emphasize the advantages of shifting towards more locally produced, plant-based food and feed, especially in nature-based proteins. Embracing an MD-aligned food system would concurrently tackle the challenges of public health, food security, resilience, and environmental sustainability. These findings hold global significance for countries confronting similar issues in reconciling nutritional recommendations with food security needs. Future strategies should concentrate on policy reforms, agricultural investments, and public involvement to strengthen Israel’s food sovereignty and nutritional landscape.

**Supplementary Information:**

The online version contains supplementary material available at 10.1186/s13584-026-00748-1.

## Background

The Food and Agriculture Organization (FAO) defines Food Systems (FS) as the multiple ecosystems, actors, and activities involved in the production, aggregation, processing, distribution, consumption, and disposal of food items and their ingredients [1]. A sustainable FS, integral to the United Nations (UN)’s Sustainable Development Goals (SDGs), under Goal 2 (Zero Hunger) and Goal 12 (Responsible Consumption and Production), emphasizes the establishment of systems that are efficient, inclusive, resilient, and sustainable, and culturally sensitive.

The current global context has significantly intensified the urgency for food system resilience. Recent global disruptions, including the COVID-19 pandemic, climate-related extreme weather events, and ongoing geopolitical conflicts, have exposed vulnerabilities in international food supply chains [2, 3]. The Russia-Ukraine conflict, for example, has disrupted global grain and fertilizer markets, impacting food security worldwide and highlighting the risks of over-dependence on imports [3]. These events emphasize developing resilient, locally adapted food systems that endure external shocks while meeting nutritional goals.

While the global food system has demonstrated overall resilience to date, it remains exposed to disruptions from geopolitical tensions, supply chain interruptions, and - most critically - the chronic, structural pressures of climate change [4]. These shocks, though often temporary at the aggregate level, generate recurrent price volatility, localized shortages, and disproportionate impacts on import-dependent and low-income populations [4]. This sensitivity is especially pronounced in areas facing complex security challenges, where ensuring stable food supplies requires careful planning and strong contingency measures. Research has shown that disruptions in food systems can trigger cascading effects on public health, economic stability, and social well-being [4]. The COVID-19 pandemic further illustrated how global supply chain disruptions can rapidly impact food availability and affordability, particularly for vulnerable populations [5].

FSs should be anchored in the One Health Approach, which considers the interconnected health of humans, animals, plants, and the environment. Complementing these principles and ensuring a positive impact on the One Health system, the Mediterranean Diet (MD) was selected as the benchmark for FSs. MD is renowned for its sustainability and health benefits, including reduced risk of cardiovascular diseases, certain cancers, and overall mortality [6, 7]. The MD is rich in plant-based foods (cereals, fruits, vegetables, legumes, tree nuts, seeds, and olives) with moderate-to-high consumption of fish and seafood, moderate consumption of eggs, poultry, and dairy products (cheese, milk, and yogurt) and low consumption of red meat, with extra-virgin olive oil used as the principal source of added fat [8].

Since 2013, the MD has been recognized by UNESCO for its intangible cultural heritage, encompassing traditional agricultural, fishing, and culinary practices that promote health and enhance cultural identity and biodiversity, making it a model diet for sustainable food systems [9]. Herforth et al., in their global review of food-based dietary guidelines (FBDGs), showed that a basic set of key principles based on the MD are “nearly universally across countries,” as well as following World Health Organization (WHO) global guidance [10]. Most FBDGs do not align with global environmental targets, especially greenhouse gas emissions reduction targets [11].

Like many other Mediterranean countries, the Israeli population’s dietary habits are significantly and substantially removed from the Mediterranean Diet’s (MD) characteristics. This shift has profound implications for food availability in Israel, which can be assessed through the Food Balance Sheet (FBS). FBS is an index developed by the FAO of the United Nations (UN) in collaboration with member nations to estimate food supply at the national level. The FBS tracks various food items for food and feed, including both primary commodities (e.g. wheat, rice, fruit, vegetables) and several processed commodities (e.g. vegetable oils, butter). It provides a comprehensive picture of the national food and feed supplies, the food loss during storage and transportation, and a country’s per capita supply patterns, showing the sources of supply. The FBS covers production, trade, feed and seed, waste, other utilization, availability, and quantities [12, 13].

Food security refers to a situation where all people, at all times, have physical, social, and economic access to sufficient, safe, and nutritious food that meets their dietary needs and food preferences for an active and healthy life. Food sovereignty, on the other hand, emphasizes people’s right to define their own food and agriculture systems, promoting sustainable, culturally appropriate food production through local farmers [14]. Combining these elements, the FBS helps detect a country’s food security status, highlighting its reliance on imported crops and foodstuffs and contributing to global exports.

Israel, located along the Mediterranean coast, has historically shared many culinary traditions with the MD. However, the influx of newcomers from other global regions, the modernization of food systems, and changing dietary habits have led to deviations from traditional Mediterranean eating patterns [15]. Recent studies indicate that only a small percentage of the Israeli population fully adheres to the MD, with a trend towards increased consumption of ultra-processed foods and a decline in the intake of fruits, vegetables, and whole grains [16]. The need for better planning of the Israeli FS to align with MD principles is evident, particularly given the current global context of supply chain vulnerabilities and climate change impacts.

Despite its geographical location, Israel faces challenges in maintaining a food environment that supports adherence to the MD. Urbanization, changing work patterns, and the influx of global food trends have contributed to a shift away from traditional eating habits [15]. Simultaneously, food security and sovereignty have become increasingly important considerations in public health and national policy, especially in countries prone to emergencies like Israel. The Import Dependency Ratio (IDR) is a key indicator of a country’s reliance on imported food products, with implications for food security, economic stability, and environmental sustainability [17]. All countries face global price dynamics in interconnected food systems. However, high import-dependency for basic staples and animal feed amplifies vulnerability. In Israel, where ~ 96% of cereals and the majority of feed and several key protein sources are imported, domestic availability and prices are particularly exposed to international market shocks, supply chain interruptions, and cost surges [18, 19]. Long-distance food transportation contributes to increased carbon emissions. It undermines the country’s efforts to align with the global agenda [20]. Moreover, local food production helps maintain traditional culinary practices and biodiversity [21].

Israel’s 2018 national dietary guidelines are explicitly grounded in Mediterranean dietary principles and are presented visually as the Israel’s New National Nutrition Recommendations - the New Nutritional Rainbow [22, 23] (replacing the earlier food pyramid). These guidelines, which emphasize plant-based foods, whole grains, legumes, olive oil, and moderate fish consumption while limiting red meat and ultra-processed foods, are integrated into school-based nutrition education curricula nationwide. This policy foundation demonstrates that Mediterranean dietary patterns are not merely theoretical constructs for Israel but are embedded in official nutrition policy, providing a strong basis for aligning agricultural production and trade policies with these health-promoting dietary recommendations. By analyzing these two perspectives, we seek to provide a comprehensive understanding of the current state of the Israeli FS, its strengths, and areas for improvement. This assessment contributes to the growing literature on sustainable and healthy FS while offering actionable insights for policymakers and public health. The findings inform strategies to better align the food system with MD principles and enhance food sovereignty, ultimately promoting public health and sustainability.

This study aims to evaluate Israel’s food system through the lens of Mediterranean Diet compliance, examining the relationships between current dietary patterns, import dependency, and food system resilience. Specifically, we: (a) quantify Israel’s import dependency ratios across key food groups; (b) compare current food availability patterns with Mediterranean Diet recommendations; and (c) model scenarios for enhancing food security and system resilience through strategic alignment with Mediterranean dietary principles.

## Methods

A comprehensive academic search was conducted to assess MD characteristics and their impact on health outcomes. The engines used are PubMed, Semantic Scholar, EBSCO Discovery, and Google Scholar. Studies from the past 10 years with the key keywords “Mediterranean diet” and “meta-analysis” (see Additional file 1 Table 1S) were included. The recommended intake is based on Israel’s New National Nutrition Recommendations - the New Nutritional Rainbow [23] and the Healthy Diets from Sustainable Food Systems Food Planet Health - EAT LANCET [24].

We selected national dietary guidelines from Greece [25], Italy [26], and Spain [27] as Mediterranean Diet reference points based on: geographic and cultural representation of traditional Mediterranean dietary patterns; availability of recent, evidence-based national guidelines; and active research and policy communities focused on Mediterranean dietary traditions. These were compared with Israel’s New National Nutrition Recommendations - the New Nutritional Rainbow [23]. Our systematic literature search revealed significant variations in research focus across Mediterranean countries. The search yielded 959 studies related to Spain, 853 for Italy, 453 for Greece, and notably fewer − 77 for Israel. This disparity highlights a potential gap in region-specific research that could inform policy decisions (Additional file 1: Table 1S: Specific search terms and respective yielded results).

The further selection and reduction process was then manually carried out by assessing the relevance of each item to the issues, first by examining the title and abstract of each article and then by delving into its detailed content.

Inclusion criteria: studies focusing on MD and related outcomes; meta-analyses and systematic reviews; studies examining food security, sustainability, or environmental impacts; and research specific to Mediterranean region countries.

Exclusion criteria: Studies older than 10 years, non-English language publications, case studies with sample size < 100, and studies without clear methodology.

In the final writing of this review, only 1–2 items were chosen as examples for each point of argument, which were deemed most for the case in point. Additionally, to explore the origins and development of the MD concept, attention was given to locating the publications of individual researchers who pioneered the field, such as Trichopoulou, Keys, and Willett. Over 250 items were examined manually, and about 50 articles were included in the final review.

**Food System Analysis Methodology** To examine Israel’s food system, we analyzed several components. First, we assessed food availability as reported by the Israeli Central Bureau of Statistics (CBS) to the Food and Agriculture Organization. We assessed its ability to allow compliance of the Israeli population with MD. We then analyzed food sovereignty by assessing local production capacity and calculating the import dependency index. The Israeli agricultural production data were drawn from the CBS for 2021 [28, 29]. Import dependence was calculated by converting product quantities from tonnes to grams per person per day. The calculation multiplied the annual quantity by 10 million (approximate population) and divided by 365 days. This is the gross import amount and does not include wear and tear, depreciation, etc. The net for each product is calculated using a similar gross vs. net inventory ratio for each food. The retrieval calculated as net for each food was calculated from the import. The percentage of availability compliance out of MD Recommendation was calculated using the following: *(Actual supply availability*100%)/MD Recommendation. Percentage of Import Compliance Out Of MD Recommendation by the following: (Imported* 100%)/MD recommendation. Descriptive analyses were provided for all food groups reported.

### Calculation of import dependency ratio (IDR) and per capita availability

Import dependency ratio (IDR) is calculated as: IDR (%) = (Gross Imports/Actual Supply Availability) × 100.

Per capita daily availability is calculated from CBS annual trade data: g/capita/day = (Annual imports in tonnes × 1,000,000 g/tonne)/(Population × 365 days).

## Worked example: legumes

To illustrate the calculation methodology, we present a detailed example for legumes corresponding to data presented in Table 2:

Given data (2021):

- Israeli population: ~10,000,000.
- Per capita daily availability from imports (from Table 2, Column C): 12.7 g/day/person.
- Total per capita daily availability (from Table 2, Column B): 24.9 g/day/person.
- Import Dependency Ratio (from Table 2, Column D): 51%.

Calculations:

1. Annual legume imports (converting from daily per capita to annual tonnes):
  o 12.7 g/day/person × 10,000,000 people × 365 days = 46,355,000,000 g.
  o = 46,355 tonnes annual imports.
2. Daily per capita availability from domestic production:
  o Total availability - Imports = 24.9–12.7 = 12.2 g/capita/day from domestic production.
3. Annual domestic legume production (converting from daily per capita to annual tonnes):
  o 12.2 g/day/person × 10,000,000 people × 365 days = 44,530,000,000 g.
  o = 44,530 tonnes annual domestic production.
4. Total annual availability:
  o 46,355 + 44,530 = 90,885 tonnes.
5. Verification of Import Dependency Ratio (IDR):
  o (46,355 tonnes/90,885 tonnes) × 100 = 51%.
  o Alternatively: (12.7 g/day/24.9 g/day) × 100 = 51%.
6. Compliance with MD recommendations:
  o MD recommendation (Table 2, Column A): 75 g/day/person.
  o Actual availability: 24.9 g/day/person.
  o Compliance rate: (24.9/75) × 100 = 33.2% (as shown in Table 2, Column E).

This example demonstrates that Israel imports 51% of its legume supply (46,355 tonnes annually), with domestic production contributing 49% (44,530 tonnes annually). However, total legume availability (24.9 g/capita/day) meets only 33% of the Mediterranean Diet recommendation of 75 g/day.

Note on net availability: These calculations represent gross availability. Net availability accounting for waste, storage losses, and non-food uses would be lower. The net-to-gross ratio varies by food group and is estimated separately for each commodity based on FAO waste coefficients.

An important caveat regarding grain imports: while total grain volumes are reported in aggregate, a substantial portion of imported wheat and other grains serves as raw material for snack foods, baked goods, and other ultra-processed products manufactured in Israel’s food industry. These products, though made from imported grains, do not align with Mediterranean Diet principles, emphasizing whole grains and minimally processed foods. Our analysis of import dependency reflects total grain flows but does not disaggregate between whole grains for MD-compatible staples versus grains destined for ultra-processed food manufacturing.

## Data sources and time frames

This analysis integrates two complementary data sources with distinct temporal coverage:

1. **FAO Food Balance Sheets (2010–2020)**: Used for trend analysis and international comparative context. These data provide longitudinal perspectives on Israel’s food system evolution and allow comparison with global patterns.
2. **Israel Central Bureau of Statistics (CBS) production and trade data (2021)**: Used for detailed import dependency ratios and food availability calculations. This represents the most recent national-level snapshot available at the time of analysis.

### Important note

Import dependency ratios and food availability calculations presented in Table 2 and throughout the main results refer to the 2021 CBS data unless otherwise specified. Historical trends referenced in the Discussion draw on the 2010–2020 FAO time series.

## Ethical considerations

This study used publicly available aggregated data and did not involve research on human subjects. Therefore, ethical approval was not required.

## Results

The analysis of Israel’s food system reveals a complex and multifaceted landscape of food availability, IDR, and MD nutritional compliance. One of the initial observations is that Israel has a higher total available calories per capita than numerous developed countries, including Australia, Canada, France, Germany, Japan, and the United States. Grains and their derivative products emerge as the primary sources of vitamins and minerals per capita.

Although Israel’s food supply includes many components of the MD, there are some notable gaps. The high availability of fruits, vegetables, and olive oil aligns well with MD principles. However, the oversupply of meat (especially poultry), dairy products, and insufficient legumes diverges from the ideal MD pattern.

The environmental cost of importing animal feed is particularly noteworthy, as it indirectly contributes to the emissions associated with Israel’s domestic meat and dairy production.

Table 1 presents weekly food intake per capita, as recommended by three Mediterranean countries: Greece, Italy, and Spain (columns A-C). The food groups in the table are divided, according to the nutritional recommendations of MD, into foods that should be consumed daily or weekly. The table reveals marked differences between the dietary recommendations of the countries observed, especially concerning the daily foods consumed. The table also exposes significant differences between professional recommendations embodied in the literature review and national recommendations. Of the three nations surveyed, Spain and Italy are most closely aligned with evidence-based recommendations derived from meta-analyses and systematic reviews of MD health outcomes.

**Table 1:** Weekly food group intake from dietary recommendations of three Mediterranean countries (kg)

		A	B	C
		Greece 2014 [26]	Italy 2018 [27]	Spain 2022 [28]
Daily	Cereals	4.2–6.7	1.7–3.4	1.2–3.4
	Vegetables	4.2–5.6	2.8–4.2	3.2–4.2
	Fruits	3.4–5.6	3.2	1.7–4.2
	Olive oil	0.42–0.52	0.14–0.28	0.14–0.21
	Dairy products	2.1–2.8	2.6	2.6–3.9
	Nuts	---	0.21–0.24	0.14–0.21
weekly	Potatoes	0.36–0.45	0.4	---
	Legumes	0.45–0.6	0.45	0.2–0.3
	Fish	0.3–0.45	0.3–0.45	0.37–0.45
	Poultry	0.12–0.3	0.2–0.3	0.3–0.37
	Eggs	1–4	2–4	1–4
	Red Meat	0.15	0.1–0.2	0.1–0.13

Values represent recommended weekly intake ranges per person according to national dietary guidelines. Countries were selected as representative examples of Mediterranean dietary patterns: Greece (origin of Mediterranean Diet concept), Italy (strong Mediterranean dietary research base), Spain (recent evidence-based guidelines). Serving sizes are defined according to each country’s national guidelines. Dash (---) indicates no specific recommendation provided. Sources: Greece - Hellenic Health Foundation (2014) [25]; Italy - CREA (2018) [26]; Spain - AESAN (2022) [27].

Analysis of dietary recommendations across Mediterranean countries reveals significant variations in the interpretation and implementation of MD principles. Spain [27] and Italy [26] align closely with professional recommendations. These perceived discrepancies emphasize the lack of uniformity in the conception of the “Mediterranean Diet” among the different nations of the region.

Table 2 presents the Israeli food supply’s compliance with MD principles based on the FBS from the Israeli CBS (2021) [28, 29]. The recommended intake is based on Israel’s New National Nutrition Recommendations - the New Nutritional Rainbow [23] and the Healthy Diets From Sustainable Food Systems Food Planet Health - EAT LANCET [24].

**Table 2:** Israeli food supply’s compliance with MD principles (based on 2021 CBS data)

Food groups	A	B	C	D	E	F
	MD Recommended intake avg. (g/day/person)	Israel’s per-capita net food supply availability 2021* (g/day/person)	Imported (g/day/person)	Import of actual availability (C*100%)/B In %	Availability compliance out of MD recommendation (B*100%)/A In %	Import dependency: % of MD recommendation met by imports (C*100%)/A In %
Whole grains, Rice, wheat, corn, and other	232	438.4	396	90.33%	189%	170.7%
Tubers or starchy vegetables: Potatoes and cassava	50	117	17.2	14.70%	234%	34.4%
Vegetables	300	400.7	50.6	12.63%	133.50%	16.9%
Fruits	200	394.8	127.7	32.35%	197%	63.9%
Dairy foods: Whole milk or equivalents	250	491.3	45.5	9.26%	196.50%	18.2%
Protein Sources						
Red Meat	14	46.1	26.5	57%	329%	189.3%
Chicken and other poultry	29	137	0.75	1%	472%	2.6%
Eggs	13	44.5	2.7	6%	342%	20.8%
Fish	28	24.2	21.1	87%	86%	75.4%
Legumes	75	24.9	12.7	51%	33.20%	16.9%
Nuts	50	36.3	29.6	82%	72.60%	59.2%
Added fats Unsaturated oils	40	87.5 **	56.5	65%	218.80%	141.3%
Saturated oils	11.8	2.7 ***	1.6	59%	22.90%	13.6%

* Net food after other uses and waste

**(Veg. oils)

***Butter CBS: Central Bureau of Statistics

Column A: Mediterranean Diet recommended intake based on EAT-Lancet Commission [25] and Israel's Nutritional Rainbow guidelines [23]

Column B: Actual per capita net food supply in Israel (2021) after accounting for waste and non-food uses

Column C: Gross imports per capita per day

Column D: Percentage of actual availability that is imported

Column E: Percentage of MD recommendation met by actual availability

Column F: Percentage of MD recommendations that could be met by imports alone

Israel’s food supply reveals a paradoxical pattern: oversupply of animal products alongside undersupply of plant-based MD staples. Poultry, eggs, and red meat exceed MD recommendations by 372%, 242%, and 229% respectively, while legumes reach only 33% of recommended intake and nuts 73%. This imbalance reflects a food system optimized for animal protein production rather than MD principles.

The country demonstrates high import dependency across several critical food groups: cereals (90% of actual availability imported), red meat (57% of actual availability imported), fish (87% of actual availability imported), legumes (51% of actual availability imported), nuts (82% of actual availability imported), and added fats (65% of actual availability imported).

Among legumes, chickpeas represent a notable exception to import dependency: Israel produces approximately 40% of domestically consumed chickpeas, reflecting both the crop’s suitability to local growing conditions and its cultural centrality to Middle Eastern cuisine. This partial self-sufficiency in a protein-rich, drought-resistant legume demonstrates the feasibility of aligning agricultural production with both Mediterranean dietary patterns and regional food traditions.

These import patterns create cascading vulnerabilities. While Israel achieves near self-sufficiency in poultry (99% domestic production), this production depends almost entirely on imported grain feed. Similarly, high dairy production relies on imported feed, creating indirect import dependency that amplifies exposure to global market disruptions. Conversely, vegetables, fruits, and potatoes demonstrate genuine domestic resilience through local production with minimal import reliance.

## Discussion

This study examined Israel’s food system through the lens of the Mediterranean Diet, focusing on security, sovereignty, resilience, and sustainability. Our analysis revealed several key findings:

### Food security challenges

high import dependency for critical foods, vulnerability to supply chain disruptions, and limited domestic production capacity.

Imports in Israel are not a fixed parameter but rather reflect current consumption patterns. If diets shift toward a Mediterranean pattern with fewer ultra-processed, animal-intensive foods and more legumes, vegetables, fruits and whole grains, trade flows will naturally adjust. Our analysis therefore represents a static picture of today’s import structure, not a prediction of inevitable future patterns. Policy interventions that reshape demand through education, pricing mechanisms, and food environment modifications are essential complements to strategies for increasing strategic domestic production.

This pattern suggests potential opportunities for optimization of the food supply system to better align with MD recommendations while considering food security and sustainability implications.

### Food sovereignty and import dependency

heavy reliance on international markets, limited control over key food supplies, and dependency on imported animal feed.

The high volume of grain imports must be interpreted with caution: while these imports could theoretically support increased whole grain consumption aligned with Mediterranean Diet recommendations, substantial portions are actually transformed into ultra-processed snack products inconsistent with MD principles. This highlights a misalignment between import composition and desirable dietary patterns, suggesting that trade policy and food industry regulation should prioritize imports and domestic production supporting whole-food Mediterranean dietary patterns over raw materials for ultra-processed food manufacturing.

### System resilience under crisis scenarios

strong in some areas (vegetables, fruits), weak in others (cereals, proteins), and the need for diversification. The domestic production of chickpeas illustrates that not all protein-rich plant foods require high import dependency. Expanding cultivation of culturally embedded, regionally adapted legumes can simultaneously advance nutritional goals, enhance system resilience, and support food sovereignty by building on existing agricultural knowledge and consumer acceptance.

### Environmental and nutritional sustainability

high carbon footprint from imports, resource intensity of current production, and opportunities for improvement.

Increasing local production is not automatically synonymous with climate resilience or sustainability. Israel faces significant resource constraints (water scarcity, limited arable land) and high climate vulnerability. Our recommendation for strategic local production focuses specifically on shifting toward less resource-intensive, MD-compliant crops - particularly plant-based proteins such as legumes - which require fewer inputs than the current animal production system that relies heavily on imported feed [30]. Any expansion of local production must be evaluated against comparative advantage principles and resource efficiency metrics, ensuring that domestic food security gains do not come at disproportionate environmental or economic costs.

The MD has been associated with numerous positive health outcomes, making it an attractive model for improving public health in Israel. Studies have consistently shown that adherence to the MD is linked to reduced risks of cardiovascular disease, certain cancers, and cognitive decline [31–33]. The diet’s high content of antioxidants, fiber, and healthy fats work synergistically to promote overall health and well-being [34]. Furthermore, the MD has been associated with lower mortality rates and increased longevity [33]. Its beneficial effects extend to improving inflammation markers and overall metabolic function [34]. The diet has also been linked to positive outcomes in prenatal health and fetal development [35], and mental health, including reduced symptoms of anxiety and depression [36].

Adopting a more MD-aligned food system could enhance Israel’s resilience to global supply chain disruptions, emergencies, and climate change impacts [37, 38]. The MD’s emphasis on diverse plant-based foods, locally adapted crops, and moderate animal product consumption creates a more flexible and robust food supply [37]. Comparative studies have shown that the MD has a lower carbon footprint, utilizes less land resources, and is more efficient in water and energy resources than other western diets [39–41]. The diet’s reliance on plant-based and local food sources makes it more sustainable and ecologically friendly over the long term [41].

Global disruptions have exposed vulnerabilities in international food supply chains, with diversified, locally-adapted systems demonstrating greater resilience [42, 43]. Israel’s current high import dependency makes it particularly vulnerable to such disruptions, as evidenced by supply chain vulnerabilities and food security challenges documented during the COVID-19 pandemic and the Russia-Ukraine conflict, which highlighted Israel’s particular exposure to global disruptions [2, 3, 44–46].

However, the environmental benefits of the MD can be compromised when foods are transported long distances. Imported animal products carry significantly higher carbon footprints than local alternatives: imported beef generates approximately 27 kg CO₂e per kilogram versus 2.5 kg for local beef, while imported salmon produces 8.4 kg versus 2.2 kg for local sources [47]. In contrast, plant-based foods like olive oil have considerably lower footprints (−5.5 to −2.7 kg CO₂eq/kg), with olive groves functioning as carbon sinks [48].

These calculations must be contextualized within Israel’s circumstances. Most irrigation water is desalinated using imported energy, meaning domestic food production carries indirect carbon costs from desalination and water distribution [49–51]. Without concurrent investment in renewable energy and efficient desalination, expanding local production could shift rather than reduce emissions. Our recommendations for increased MD-compliant domestic production must therefore prioritize drought-resistant legumes and vegetables requiring less irrigation than grain-intensive animal feed, coupled with transition to low-carbon energy sources [30, 51].

Such data underscore the importance of promoting local food consumption, especially for animal products, as an effective measure to reduce environmental pressures like greenhouse gas emissions, water use, and land footprint [52]. Additionally, considering the broader environmental footprint of food commodities - such as cropland, blue water, and nutrient applications - highlights the need for systemic changes in sourcing and dietary choices to support sustainable food systems [47, 52]. Israel’s dependence on imported infant formula represents a critical vulnerability for the most nutritionally vulnerable population, though promoting breastfeeding remains the primary public health strategy for infant nutrition resilience.

Comprehensive strategies must address immediate nutritional needs, long-term food security, and resilience goals.

Our proposal should not be seen as a call for autarky. Radical self-sufficiency would mainly benefit countries with abundant land and water resources while worsening global food inequalities [18, 53]. What we support is resilience-focused rebalancing: increasing the share of key Mediterranean diet foods that Israel can reliably produce domestically, while maintaining trade connections and, where geopolitically possible, creating regional cooperation frameworks. Not every country can reach similar levels of domestic production, which highlights the importance of international cooperation, solidarity mechanisms, and fair trade rules for global food security.

Our findings indicate that transitioning towards more plant-based protein sources that can be locally produced, such as legumes, could substantially reduce both import dependence and the overall carbon footprint of the Israeli diet.

## Efficiency trade-offs and global implications

Our scenarios should not be interpreted as advocating for global autarky. A world where every country produces all food domestically would result in higher prices, reduced efficiency, diminished competition, and increased inequality, with greatest harm to resource-poor economies [54]. Food security would become zero-sum competition, undermining global welfare.

What we propose for Israel is strategic diversification - not “100% self-sufficiency” but developing domestic capacity for key Mediterranean-diet foods while maintaining trade relationships and, where feasible, regional cooperation frameworks. Smart interdependence remains the primary goal; our national scenarios serve as contingency plans for severe disruptions, not blueprints for autarkic systems [54, 55]. The economic principles of trade - comparative advantage, economies of scale, competitive markets - remain essential. Our analysis intentionally focuses on individual nations without estimating welfare costs of widespread self-sufficiency, which would actually reinforce the case for regional cooperation as the primary long-term approach.

Enhancing local production and consumption of legumes, nuts, and whole grains could better align Israel’s food system with MD recommendations, potentially improving health outcomes and environmental sustainability.

## Policy recommendations

Based on our findings, we propose comprehensive policy recommendations for Israeli policymakers and stakeholders to strengthen the resilience of the food system while aligning with MD principles. Israel’s food system challenge is characterized by surplus rather than scarcity: current consumption levels exceed nutritional requirements, and food waste remains substantial. Our scenarios are intentionally conservative because they are calibrated to today’s inflated consumption and loss patterns, not to the lower intake and waste levels compatible with health and sustainability goals [22, 23]. In practice, reducing overconsumption and food waste represents a first-line resilience strategy that could significantly enhance food security without expanding production. Because our calculations are based on current apparent consumption, they implicitly include overconsumption and waste, thereby overstating the quantities required for a healthy, sustainable diet.

The alignment between agricultural policy and Mediterranean Diet principles is not only epidemiologically sound but also policy-coherent: Israel’s 2018 national dietary guidelines already endorse Mediterranean dietary patterns through the Nutritional Rainbow framework used in schools and public health messaging. Current food system structure and import patterns, however, do not adequately support these official nutritional recommendations, creating a disconnect between health policy and food system reality that policy interventions should address.

**National level recommendations**:

1. Develop a National Food Security Strategy that prioritizes local production of MD-compliant foods, especially legumes, nuts, and whole grains, to decrease import dependency and strengthen food sovereignty [56].
2. Implement agricultural diversification policies to enhance domestic production of plant-based proteins, particularly legumes, by providing targeted subsidies and research support for drought-resistant varieties [57].
3. Establish strategic food reserves for critical imported foods, such as cereals and fish, to buffer against supply chain disruptions, while developing long-term substitution strategies [55].
4. Re-orient water-food governance to leverage existing capabilities: Building on Israel’s world-leading water recycling infrastructure and precision irrigation technologies, prioritize allocation of desalinated and reclaimed water to Mediterranean-diet crops (legumes, vegetables, fruits, whole grains) and to vulnerable agricultural regions. Implement transparent allocation rules that protect small and medium producers and discourage water allocation to water-intensive, low-nutrition crops. Focus policy on strategic governance of existing technologies rather than technological development [51, 58].
5. Develop climate-resilient agriculture programs that help farmers adopt sustainable practices suited for Mediterranean climates, such as precision agriculture and regenerative farming methods [59].

### International applications

These recommendations have wider applicability to other Mediterranean countries and regions facing similar challenges:

1. Mediterranean Cooperation Framework: Countries in the Mediterranean basin could benefit from regional collaboration in developing climate-adapted crop varieties and sharing sustainable agricultural practices [60].
2. Small Island Developing States (SIDS): The framework for balancing nutritional guidelines with concerns about food security is especially pertinent for island nations that have high import dependencies [61].
3. Arid and Semi-Arid Regions: The strategies for water-efficient agriculture and approaches for developing drought-resistant crops are relevant to countries around the world facing similar climatic challenges [62].
4. Post-Conflict Regions: Emphasizing food sovereignty and the development of local production capacity is particularly important for countries recovering from conflicts and striving to rebuild their food systems [63].

The policy framework created for Israel can act as a model for other countries aiming to align their food systems with healthy dietary patterns while also ensuring food security and environmental sustainability.

### Limitations

This study has several important limitations that should be acknowledged:

### Data and methodological limitations

- The study relies significantly on Food Balance Sheets and import/export data, which might not always accurately represent actual consumption patterns at the household level [53].
- The data represent a specific period (2010–2020) and may not capture recent changes in dietary patterns or food production systems, particularly those occurring after the COVID-19 pandemic [64].
- The Mediterranean Diet recommendations, used as benchmarks, vary across different countries and scientific sources, which may impact the accuracy of compliance assessments [65].

Our modelling has additional important limitations. First, our calculations are based on current apparent consumption and availability data and therefore implicitly include overconsumption and avoidable food waste. As a result, the nutritional “needs” we model exceed requirements for healthy, sustainable diets, making our independence targets conservative. Future work should model scenarios based on recommended intake levels aligned with MD principles and realistic waste reduction targets, which would likely show that lower levels of domestic production and imports could meet actual nutritional requirements.

Second, we focus on production and imports for domestic consumption and do not explicitly model exports by food group. While Israel is a net importer for most staples, it does export certain high-value products (fruits, vegetables, specialty crops). Incorporating full net trade perspectives and the option of diverting export-oriented production to domestic markets during crises would likely improve resilience estimates and should be addressed in future quantitative work.

Third, we consider only food commodities and do not account for land and water currently allocated to non-food agriculture (e.g., ornamental crops, cut flowers). These represent an additional pool of resources that could, in principle, be reallocated toward food production in extreme scenarios. Future scenario planning should quantify this potential reserve capacity and assess the feasibility and costs of such reallocation.

Finally, our analysis is national by design and does not model the global welfare costs that would arise if many countries simultaneously pursued maximal self-sufficiency. Fully internalizing such efficiency and competition losses would likely strengthen the case for regional and global cooperation as the primary long-term solution, with national diversification serving as a bounded resilience buffer rather than an isolationist strategy.

### Scope and context limitations

- The study does not consider socio-economic and demographic factors that may influence dietary choices and food access among various population segments in Israel [66].
- Cultural and religious dietary preferences specific to Israel’s diverse population were not thoroughly incorporated into the analysis [67].
- The analysis does not include a comprehensive life-cycle assessment of various food items, which would offer a more complete picture of environmental impacts [62].

### Stakeholder and implementation limitations

- The study lacks direct input from key stakeholders, including consumers, policymakers, farmers, and food industry representatives, which could provide valuable context for the findings [63].
- The recommendations may not fully address practical implementation challenges, including economic constraints, political considerations, and existing agricultural infrastructure [64].

### Generalizability limitations

- While the systematic approach can be applied to other regions, the specific findings are tailored to Israel’s unique climate, geopolitical situation, and cultural context, and may not directly translate to other countries [68].
- The study does not address potential trade-offs between various policy objectives, such as food security versus environmental sustainability, which may necessitate different prioritizations in different contexts [54, 69].

Despite these limitations, the study offers valuable insights into the complex relationships among dietary guidelines, food security, and sustainability, providing a framework that can be adapted for use in other Mediterranean and similar contexts worldwide.

## Conclusion

The current global context of supply chain vulnerabilities, climate change impacts, and geopolitical tensions underscores the critical importance of developing resilient, locally adapted food systems. Our analysis of Israel’s food system reveals both significant challenges and opportunities in achieving food security while promoting health and environmental sustainability.

### Key findings

Israel demonstrates high import dependency across critical food groups (90% cereals, 87% fish, 65% added fats), creating vulnerability to supply chain disruptions.

### Strategic implications

Transitioning to a more MD-aligned food system could enhance Israel’s food sovereignty and resilience to global supply chain disruptions while simultaneously improving population health and reducing environmental impact. The alignment between Israel’s 2018 national dietary guidelines (the Nutritional Rainbow) and Mediterranean dietary principles provides a strong policy foundation for this transition.

**Policy recommendations**:

1. Increase local production of MD-compliant foods, particularly legumes, nuts, and whole grains.
2. Reduce import dependency through strategic agricultural development focused on culturally embedded, regionally adapted crops (e.g., expanding chickpea production beyond current 40% self-sufficiency).
3. Implement policies to encourage MD-aligned food supply patterns through agricultural subsidies, food industry regulation, and public education.
4. Develop resilience measures including strategic food reserves and climate-adapted agriculture programs.
5. Invest in sustainable agricultural practices leveraging Israel’s existing water recycling infrastructure and precision irrigation technologies.

### Global relevance

These findings have broader implications beyond Israel’s borders. The framework developed here - examining food systems through the lens of sustainable dietary patterns while considering import dependencies and local production capacities - can be adapted for use in various global contexts, from other Mediterranean countries to small island developing states and regions with similar climatic or geopolitical challenges.

### Future research directions

**Future research should focus on**:

- Detailed resource allocation modeling for MD-compliant crop expansion.
- Quantitative waste reduction scenarios and their impact on food security.
- Export reallocation potential during crisis scenarios.
- Regional cooperation frameworks for the Eastern Mediterranean.
- Life-cycle assessments comparing domestic vs. imported food production under Israeli conditions.

In the longer term, regional cooperation in the Eastern Mediterranean represents the optimal strategy for balancing efficiency, resilience and climate goals. Our national-level scenarios should be read as preparedness stress-tests - what Israel can achieve domestically if necessary - while the normative long-term horizon remains a cooperative regional food system that leverages comparative advantages across countries while building collective resilience to shared climate and geopolitical risks.

## Supplementary Information

Supplementary Material 1.

## Data Availability

Availability of data and materialsThe datasets supporting the conclusions of this article are available in the Israel Central Bureau of Statistics repository [https://www.cbs.gov.il/en/publications/Pages/2021/Agricultural-Production-Statistics-2010-2020.aspx], the Israel Central Bureau of Statistics Food Balance Sheets repository [https://www.cbs.gov.il/en/publications/Pages/2024/Food-Balance-Sheets-2021-2022.aspx], and the FAO Food Balance Sheets repository [http://www.fao.org/faostat/en/#data/FBS].Processed datasets and analysis code are available from the corresponding author upon reasonable request.

## Abbreviations

MD: Mediterranean Diet
IDR: Import Dependency Ratios
CBS: Central Bureau of Statistics
FAO: The Food and Agriculture Organization
FS: Food Systems
FBS: Food Balance Sheet
