# Supplementary material for: Reimagining Israel’s food system: balancing mediterranean diet recommendations with national food security, sovereignty and resilience
Source: Isr J Health Policy Res. 2026 Mar 6;15:6. doi: 10.1186/s13584-026-00748-1 (PMC12964903; doi:10.1186/s13584-026-00748-1)
Supplement: Supplementary file 1 — Supplementary Material 1. [file 13584_2026_748_MOESM1_ESM.docx]

Appendix:

**Table 1S: Specific search terms and respective yielded results**

| Major terms | Specific terms | 10-years (Since 2014) |
| --- | --- | --- |
| Mediterranean diet | +Food Security | 102 |
|  | +Sustainability | 723 |
|  | +Environment | 629 |
|  |  |  |
| Mediterranean diet | + Spain | 959 |
|  | + Italy | 853 |
|  | + Greece | 453 |
|  | + Israel | 77 |
